# Development and validation of a multivariable model predicting the required catheter dwell time among mechanically ventilated critically ill patients in three randomized trials

**DOI:** 10.1186/s13613-023-01099-9

**Published:** 2023-01-16

**Authors:** Jeanne Iachkine, Niccolò Buetti, Harm-Jan de Grooth, Anaïs R. Briant, Olivier Mimoz, Bruno Mégarbane, Jean-Paul Mira, Xavier Valette, Cédric Daubin, Damien du Cheyron, Leonard A. Mermel, Jean-François Timsit, Jean-Jacques Parienti

**Affiliations:** 1 Department of Clinical Research and Biostatistics, Caen University Hospital and Caen Normandy University, Caen, France; 2 INSERM U1311 DYNAMICURE, Caen Normandy University, Caen, France; 3 Infection Control Program and World Health Organization Collaborating Center on Patient Safety, Hospitals and Faculty of Medicine, University of Geneva, Geneva, Switzerland; 4 Department of Intensive Care, Amsterdam UMC Location Vrije Universiteit Amsterdam, De Boelelaan 1117, Amsterdam, The Netherlands; 5 Inserm U1070, Poitiers University, Poitiers, France; 6 Medical and Toxicological Intensive Care Unit, Lariboisière Hospital, AP-HP, INSERM, UMRS-1144, Paris University, Paris, France; 7 Medical ICU, Cochin Hospital, AP-HP, 75014 Paris, France; 8 Department of Medical Intensive Care, Caen University Hospital, 14000 Caen, France; 9 Department of Epidemiology and Infection Prevention, Lifespan Hospital System, Providence, RI USA; 10 Department of Medicine, Warren Alpert Medical School of Brown University, Providence, RI USA; 11 Medical and Infectious Diseases ICU (MI2), Bichat Hospital, AP-HP, University of Paris, IAME, INSERM U1137, Paris, France; 12 Poitiers University Hospital, 86021 Poitiers, France

**Keywords:** Catheter dwell time, Intensive care unit, Central venous catheters, Predictive score, Critical care

## Abstract

**Background:**

The anatomic site for central venous catheter insertion influences the risk of central venous catheter-related intravascular complications. We developed and validated a predictive score of required catheter dwell time to identify critically ill patients at higher risk of intravascular complications.

**Methods:**

We retrospectively conducted a cohort study from three multicenter randomized controlled trials enrolling consecutive patients requiring central venous catheterization. The primary outcome was the required catheter dwell time, defined as the period between the first catheter insertion and removal of the last catheter for absence of utility. Predictors were identified in the training cohort (3SITES trial; 2336 patients) through multivariable analyses based on the subdistribution hazard function accounting for death as a competing event. Internal validation was performed in the training cohort by 500 bootstraps to derive the CVC-IN score from robust risk factors. External validation of the CVC-IN score were performed in the testing cohort (CLEAN, and DRESSING2; 2371 patients).

**Results:**

The analysis was restricted to patients requiring mechanical ventilation to comply with model assumptions. Immunosuppression (2 points), high creatinine > 100 micromol/L (2 points), use of vasopressor (1 point), obesity (1 point) and older age (40–59, 1 point; ≥ 60, 2 points) were independently associated with the required catheter dwell time. At day 28, area under the ROC curve for the CVC-IN score was 0.69, 95% confidence interval (CI) [0.66–0.72] in the training cohort and 0.64, 95% CI [0.61–0.66] in the testing cohort. Patients with a CVC-IN score ≥ 4 in the overall cohort had a median required catheter dwell time of 24 days (versus 11 days for CVC-IN score < 4 points). The positive predictive value of a CVC-IN score ≥ 4 was 76.9% for > 7 days required catheter dwell time in the testing cohort.

**Conclusion:**

The CVC-IN score, which can be used for the first catheter, had a modest ability to discriminate required catheter dwell time. Nevertheless, preference of the subclavian site may contribute to limit the risk of intravascular complications, in particular among ventilated patients with high CVC-IN score.

*Trials Registration* NCT01479153, NCT01629550, NCT01189682

**Supplementary Information:**

The online version contains supplementary material available at 10.1186/s13613-023-01099-9.

## Background

Short-term central venous catheters (CVC) are essential in the management of patients admitted to an Intensive Care Unit (ICU). Unfortunately, their use is responsible for complications, with a peak incidence at the time of insertion.

Ultrasound-guided internal jugular vein (IJV) insertion is easy to learn and safe with a low incidence of mechanical complications [1–3]. Therefore, it has become a standard of care in many ICUs for temporary central venous access (CVA) [4]. Compared to subclavian vein (SCV), IJV insertion is associated with a higher risk of intravascular complications [5], including catheter-related infection (CRI) [6, 7] and deep vein thrombosis (DVT). Noteworthy, the new SHEA/IDSA/APIC Compendium considered the SCV site preferable for CVC insertion in the intensive care setting to reduce infectious complications [8].

Which patients could benefit the most from a SCV site preference to reduce the risk of catheter-related bloodstream infection and symptomatic deep-venous thrombosis? In this context, the expected catheter dwell time is important because the risk of catheter-related intravascular complication associated with femoral or IJV is cumulative as the CVC exposure increases [9, 10], whereas the risk of mechanical complications associated with SCV, in particular pneumothorax, is mostly considered interventional. Moreover, the catheter dwell time is uncertain at the time of CVC insertion. For example, the correlation between estimated and actual CVC time by intensivists was low (*R*^2^ = 0.07) in a prospective cohort of 200 critically ill patients [11].

In order to guide optimal site selection, we studied the required catheter dwell time based on clinical characteristics available at CVC insertion to develop the CVC-IN score.

## Methods

### Study design

This report complies with the TRIPOD statement [12]. In this post hoc analysis, we used three completed multicenter randomized controlled trials (RCT) with prospectively collected databases. The 3SITES study [5], which investigated the impact of the anatomical insertion site on mechanical and intravascular catheter complications, was readily available to the corresponding author and therefore served as training cohort, for convenience. Subsequently, we used the CLEAN and DRESSING2 studies, which investigated different prevention strategies on the incidence of CRI, as testing cohort for external validation [13, 14].

### Study patients and catheters

Inclusion criteria slightly differed between the studies and are detailed in Additional file 1: Annex 1. All patients were adults requiring catheterization from a new venipuncture in the ICU. Data for more than one catheter per patient were allowed in the three studies. Regarding the current study, only CVCs were considered with exclusion of arterial catheters and dialysis catheters. Patients with unknown catheter dwell time or for which the catheter was not inserted after randomization were also excluded.

### Outcome and definitions

Patients were followed until day 28, ICU discharge, or death. The primary outcome was the required catheter dwell time at day 28, defined as the time from insertion of the first CVC to removal of the last CVC for absence of further utility. The decision of removal for absence of further utility was made independently by the physicians caring for each patient. Patients for whom the catheter was removed for other or unknown reasons without subsequent reinsertion of a new catheter were censored. Other possible reasons for catheter removal included: suspected CRI, accidental or systematic removal and catheter dysfunction. Patients with a required catheter dwell time > 28 days were censored at day 28. Patients discharged with their catheter were censored at the time of ICU discharge.

Some patients had several successive catheterization episodes during the ICU stay. In this situation, the respective dwell times of each catheter were summed up, until removal due to absence of further utility to calculate the required catheter dwell time. If reinsertion of a new catheter occurred more than 72 h after previous catheter removal, the patient was censored at the time of catheter removal, and the subsequent catheterization episode was not considered (Additional file 1: Figure S1).

### Statistical analysis

The unit of analysis was the patient. No computation of sample size was performed a priori. Baseline characteristics of the patients were described using numbers (percentages) for categorical variables, and median [inter-quartile range (IQR)] for quantitative variables. The statistical analysis plan had four steps, detailed in the Additional file 1: Annex 2. The number of observations with missing data was low and the imputation strategy is described in the Additional file 1: Annex 2.

We identified independent factors associated with required catheter dwell time at day 28 from univariable and multivariable Fine and Gray subdistribution-hazard models to account for death as a competing event in the training cohort [15] as recommended [16]. All variables included in the model were available at catheter insertion. The proportional hazard (PH) assumption was assessed graphically by plotting the Schoenfeld residuals (Additional file 1: Figure S2) [17]. Invasive mechanical ventilation (MV) did not verify the PH assumption, thus only patients requiring MV were analyzed. Then, we assessed internal validity by resampling methods to retain only robust risk factors and to estimate their coefficients to correct for over optimism [18]. Based on the strength of their association with the required catheter dwell time at day 28, we derived a points-based score (called “CVC-IN”), with a higher value signifying longer time to catheter removal for absence of further utility (Additional file 1: Annex 3) [19]. The external validity of the CVC-IN score was assessed in the testing cohort. In both cohorts, the discrimination was assessed using the area under 28-day time-dependent receiving operator characteristics (ROC) curves. Calibration was assessed in the testing cohort by the Brier score, with a value of 0.25 indicating poor calibration [20, 21]. The CVC-IN score was dichotomized as “low” and “high” categories using the median value in the training cohort as threshold, and sensitivity, specificity, positive predictive value (PPV) and negative predictive value (NPV) were calculated [22]. Cumulative incidence functions curves were stratified by the dichotomized CVC-in score in the training and testing cohorts, and in the overall cohort.

We performed several sensitivity analyses in the training cohort. The first replicated the main analysis for the first catheter of each patient. The second replicated the main analysis for all the catheters inserted until day 28, regardless of the time between catheter removal and reinsertion of a new catheter. Finally, we conducted univariable and multivariable Cox models for death and catheter removal for absence of further utility, to obtain cause-specific hazard ratios for each event.

All analyses were performed with SAS software V9.4 (SAS Institute, NC, Cary), and R software (R Foundation for Statistical Computing, Vienna, Austria).

## Results

### Patients characteristic

**Fig. 1:**
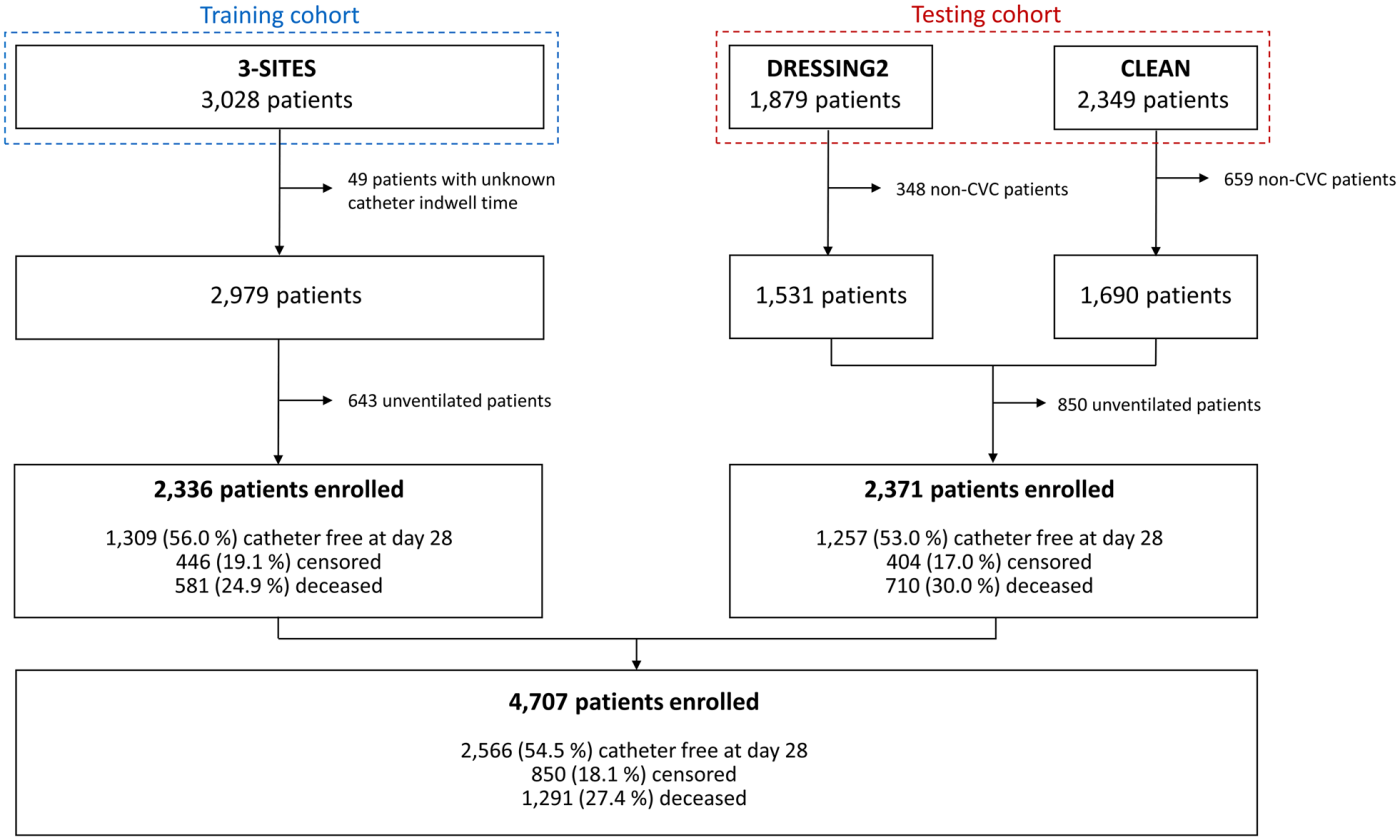
Flowchart

We included 2336 patients in the training cohort, after exclusion of 49 patients with unknown catheter dwell time and 643 non-ventilated patients (Fig. 1). Baseline characteristics are presented in Table 1. Most patients were male (65.5%) with a median age of 63 years. 498 (21.3%) patients had a body mass index (BMI) ≥ 30 kg/m^2^ and 460 (19.7%) had diabetes. Data concerning BMI were missing for 173 (7.4%) patients, and data concerning a past medical history of diabetes were missing for 253 (10.8%) patients. 244 (10.5%) patients had a past medical history of either solid or hematological malignancy or Human Immunodeficiency Virus (HIV) infection and were considered as immunocompromised. The median Simplified Acute Physiology Score (SAPS) II was 57, and 1455 (69.8%) patients required vasopressors at admission. Data concerning vasopressors were missing for 250 (10.7%) patients. 1093 (46.8%) patients had baseline creatinine levels > 100 µmol/L.

**Table 1:** Baseline characteristics of patients in the training and testing cohorts

Patients characteristics	Training cohort	Testing cohort
	*n* = 2336	*n* = 2371
Male, *n* (%)	1530 (65.5)	1589 (67.0)
Age, median [IQR]	63 [52–75]	64 [53–74]
< 40 years, *n* (%)	203 (8.7)	218 (9.2)
40–59 years, *n* (%)	774 (33.1)	700 (29.5)
60–69 years, *n* (%)	538 (23.0)	586 (24.7)
70–74 years, *n* (%)	253 (10.8)	277 (11.7)
75–80 years, *n* (%)	248 (10.6)	284 (12.0)
≥ 80 years, *n* (%)	320 (13.7)	306 (12.9)
Diabetes, *n* (%)	460 (19.7)	146 (6.2)
BMI^2^, median [IQR]	25.7 [22.5–29.3]	26.9 [23.1–30.2]
Obesity^3^, *n* (%)	498 (21.3)	622 (26.3)
Immunosuppression, *n* (%)	244 (10.5)	238 (10.0)
Body temperature ≥ 39 °C	125 (5.4)	
SAPS2^1^, median [IQR]	57 [45–72]	57 [43–72]
Vasopressors use, *n *(%)	1455 (69.8)	1292 (54.5)
Creatinine > 100 µM, *n* (%)	1093 (46.8)	1131 (47.7)

^1^*SAPS2* Simplified Acute Physiology Score

^2^BMI body mass index

^3^BMI ≥ 30 kg/m^2^

We included 2371 patients in the testing cohort after exclusion of 850 non-ventilated patients (Fig. 1). Patients were mostly male (67.0%) with a median age of 64 years. 622 (26.3%) patients had a BMI ≥ 30 kg/m^2^ and 146 (6.2%) had diabetes. BMI was missing for 213 (9.0%) patients. 238 (10.0%) patients were immunocompromised. The median SAPS II was 57 and 1292 (54.5%) patients required vasopressors. After imputation, 1131 (47.7%) patients were considered as having creatinine levels > 100 µmol/L at baseline.

### Outcomes

In the training cohort, 277 (11.9%) patients had several CVCs inserted during their stay (Additional file 1: Table S1). The median catheter dwell time was 6 days (IQR [3–10]). At day 28, 1309 (56.0%) patients were catheter-free, 581 (24.9%) died and 446 (19.1%) were censored. 82 (3.5%) patients were discharged with their catheter and censored at ICU discharge.

In the testing cohort, 382 (16.1%) patients had several CVCs inserted during their stay (Additional file 1: Table S1). The median catheter dwell time was 7 days (IQR [3–13]). At day 28, 1257 (53.0%) patients were catheter-free, 710 (30.0%) died and 404 (17.0%) were censored. 56 (2.4%) patients were discharged with their catheter and censored at ICU discharge.

### Development of the CVC-IN score

On univariable analysis, gender (male), age, obesity, diabetes, immunosuppression, creatinine > 100 µmol/L and vasopressors were associated with required catheter dwell time at day 28, as shown in Table 2. No evidence of multi-colinearity was observed (Additional file 1: Table S2) and all risk factors were introduced in the multivariable model. After backward and forward selection, age, obesity, immunosuppression, creatinine > 100 µmol/L and vasopressors were independently associated with required catheter dwell time at day 28 (Table 2). The same analysis in a five-time imputed dataset gave similar results (Additional file 1: Table S3).

**Table 2:** Univariable and multivariable subdistribution hazard models for time to catheter removal for absence of further utility in the training cohort (*n* = 2336)

Risk factors	Univariable analysis			Multivariable analysis		
	HR^1^	95% CI^2^	*p*-value	Adjusted HR^1^	95% CI^2^	*p*-value
Male	0.87	[0.79–0.97]	**0.014**			
Age			< 0.001*			< 0.001*
< 40 years	1	–	–	1	–	–
40–59 years	0.65	[0.54–0.78]	** < 0.001**	0.70	[0.58–0.85]	** < 0.001**
60–69 years	0.54	[0.44–0.66]	** < 0.001**	0.63	[0.51–0.77]	** < 0.001**
70–74 years	0.49	[0.39–0.62]	** < 0.001**	0.58	[0.46–0.74]	** < 0.001**
75–80 years	0.45	[0.36–0.57]	** < 0.001**	0.56	[0.44–0.71]	** < 0.001**
≥ 80 years	0.51	[0.41–0.63]	** < 0.001**	0.62	[0.50–0.78]	** < 0.001**
Body temperature ≥ 39 °C	0.94	[0.76–1.17]	0.60			
Diabetes	0.78	[0.68–0.90]	** < 0.001**	–	–	–
Obesity^3^	0.84	[0.74–0.95]	**0.006**	0.85	[0.75–0.96]	**0.012**
Vasopressors use	0.70	[0.63–0.78]	** < 0.001**	0.78	[0.70–0.87]	** < 0.001**
Immunosuppression	0.62	[0.51–0.75]	** < 0.001**	0.64	[0.52–0.77]	** < 0.001**
Creatinine > 100 µmol/L	0.59	[0.53–0.66]	** < 0.001**	0.66	[0.59–0.74]	** < 0.001**

^1^*HR* hazard ratio: values < 1 reflect longer catheter dwell time

^2^*CI *confidence interval

^3^Body mass index ≥ 30 kg/m^2^

*p-value is for type III effect

Bold values denote statistical significance

**Fig. 2:**
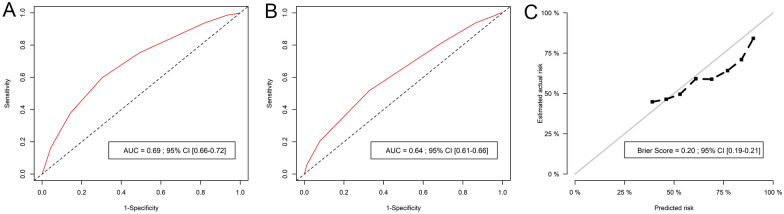
Receiver operating characteristics curves for the CVC-IN score in the **a** training and **b** testing cohorts and **c** calibration plot in the testing cohort. *CI *confidence interval

Based on 500-time repetitions of the multivariable model in bootstrap samples and subsamples, the five risk factors were considered robust (Additional file 1: Table S4). To derive the CVC-IN score as a simple points-based score, points were attached to the risk factors according to their bootstrapped coefficients. Age accounted for 0 point if < 40 years, 1 point if 40–59 years, and 2 points if ≥ 60 years. Obesity and vasopressors accounted for 1 point, while immunosuppression and creatinine > 100 µmol/L accounted for 2 points. The CVC-IN score ranged from 0 to 8 points. Using its median value as threshold, the CVC-IN score was considered “high” if ≥ 4 points and “low” if < 4 points. In the training cohort, the area under the 28-day ROC curve (AUC) was 0.69, 95% CI [0.66–0.72] (Fig. 2). The sensitivity, specificity, PPV and NPV for a CVC-IN score ≥ 4 are displayed in Table 3, for different time horizons. The cumulative incidence of catheter removal for absence of further utility and death is displayed in Additional file 1: Figure S3. While the cumulative incidence of catheter removal for absence of further utility was higher for patients with a “low” CVC-IN score, the cumulative incidence of death was higher for patients with a “high” CVC-IN score. The predicted probability of being catheter-free reached 50% (median time) at day 9 for patients with a “low” CVC-IN score and 50% at day 20 for patients with a “high” CVC-IN score.

**Table 3:** Sensitivity, specificity, positive and negative predictive values at different time horizons for CVC-IN score ≥ 4 points in the training and testing cohorts

Time horizon	Training cohort (*n* = 2,336)		Testing cohort (*n* = 2,371)	
	Value (%)	95% CI^1^	Value (%)	95% CI^1^
*7 days*				
Sensitivity	63.9	[60.4–67.4]	57.6	[53.7–61.5]
Specificity	55.3	[52.8–57.8]	54.1	[51.8–56.4]
Positive predictive value	74.2^a^	[71.5–76.9]	76.9	[74.4–79.4]
Negative predictive value	43.1	[40.3–45.9]	32.5	[29.7–35.3]
*14 days*				
Sensitivity	61.8	[58.9–64.7]	55.4	[52.4–58.4]
Specificity	61.3	[58.1–64.5]	57.3	[54.5–60.1]
Positive predictive value	57.3	[54.2–60.4]	58.7	[55.8–61.6]
Negative predictive value	65.6	[62.6–68.6]	54.0	[51.0–57.0]
*28 days*				
Sensitivity	59.9	[57.2–62.6]	55.6	[52.8–58.4]
Specificity	69.5	[66.3–72.7]	64.4	[61.7–67.1]
Positive predictive value	42.2	[39.0–45.4]	44.6	[41.5–47.7]
Negative predictive value	82.4	[80.0–84.8]	73.7	[70.9–76.5]

^1^CI confidence interval

^a^For example, the probability of needing a central-venous access for at least 7 days when the CVC-IN score is ≥ 4 points is 0.742 in the training cohort

### External validation of the CVC-IN score

In the testing cohort, the 28-day AUC was 0.64, 95% CI [0.61–0.66]. The Brier score was 0.20, 95% CI [0.19–0.21] (Fig. 2). The sensitivity, specificity, PPV and NPV for a CVC-IN score ≥ 4 are displayed in Table 3, for different time horizons. The CVC-IN score was < 4 for 1,170 (49.4%) patients, with a predicted probability of being catheter-free reaching 50% at day 13 while 1201 (50.6%) patients had a CVC-IN ≥ 4 score, with a probability of being catheter-free reaching 50% at day 28. The cumulative incidence of catheter removal for absence of further utility and death in the testing cohort is displayed in Additional file 1: Figure S4.

### CVC-IN score in the overall cohort

**Fig. 3:**
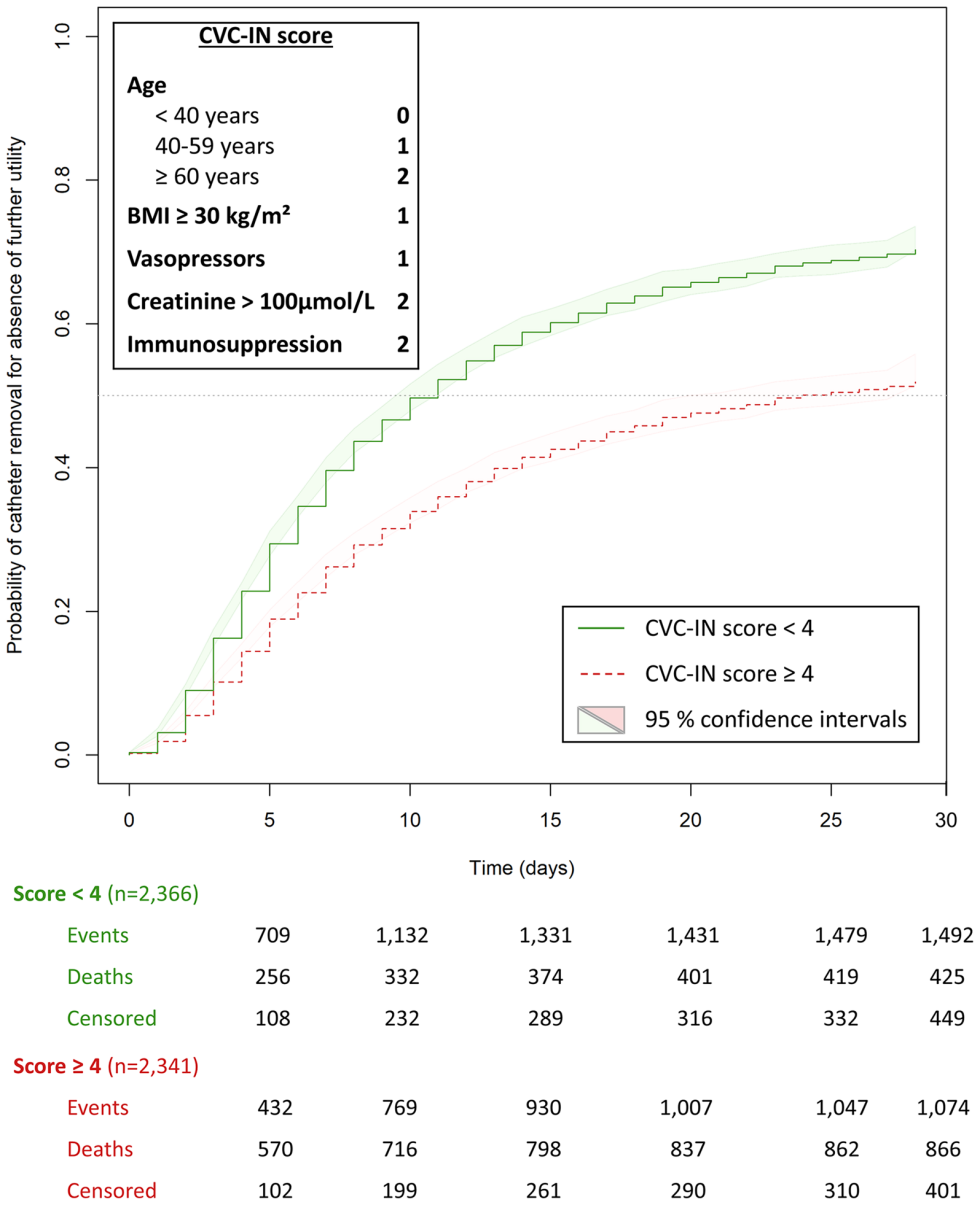
Cumulative incidence of catheter removal for absence of further utility according to the CVC-IN score risk-categories in the overall cohort (*n* = 4707). *BMI* body mass index

In the overall cohort, constituted by pooling the training and testing cohorts, 2366/4707 (50.3%) patients had a “low” CVC-IN score while 2341 (49.7%) had a “high” CVC-IN score. The median observed catheter dwell time was 6 days (IQR [3–12] in the low CVC-IN score group and 6 days (IQR [3–11]) in the high CVC-IN score group. The probability of being catheter-free was 50% at day 11 in the “low” CVC-IN score group and 50% at day 24 in the “high” CVC-IN score group (Fig. 3). The cumulative incidence of catheter removal for absence of further utility by total points is displayed in Additional file 1: Figure S5. The cumulative incidence of death according to the CVC-IN score risk group in the overall cohort is displayed in Additional file 1: Figure S6.

### Sensitivity analyses in the training cohort

Considering only the first catheter inserted in each patient, the median dwell time was 5 days (IQR [3–9]). On multivariable analysis, age, obesity, immunosuppression, creatinine > 100 µmol/L and vasopressors were independently associated with catheter dwell time. These results are consistent with the principal analysis (Additional file 1: Table S5).

In the training cohort, 77 (3.3%) patients had a catheter inserted more than 72 h after removal of the previous catheter and were censored in the principal analysis. Considering these catheters, the median catheter dwell time was 6 days (IQR [3–10]). 425 (18.2%) patients were censored, 1327 (56.8%) were catheter-free at day 28 and 584 (25.0%) died. On multivariable analysis, age, immunosuppression, creatinine > 100 µmol/L and vasopressors were independently associated with catheter dwell time. Results are displayed in Additional file 1: Table S6.

Using univariable and multivariable Cox models, we assessed the cause-specific hazard ratios for death and catheter removal for absence of further utility. On multivariable analysis, age, vasopressors, immunosuppression and creatinine > 100 µmol/L were associated with death (Additional file 1: Table S7). Of note, those factors were associated with longer catheter dwell time in the principal analysis.

On multivariable cause-specific hazard analysis, gender, age, obesity, immunosuppression and creatinine > 100 µmol/L were independently associated with longer catheter dwell time (Additional file 1: Table S7). In contrast with the principal analysis, vasopressors use was not significantly associated with catheter removal for absence of further utility on multivariable analysis.

## Discussion

High CVC-IN score, which combines immunosuppression, creatinine > 100 µmol/L, older age, obesity and use of vasopressors had a significant but moderate ability to discriminate which ventilated patients required the use of a CVC for a long period of time in the ICU, starting with the first CVC inserted in ICU. A CVC-IN score ≥ 4 could identify a subgroup of patients with a median predicted CVC requirement of 24 days, compared to 11 days for the remaining patients. We suggest this former group is more likely to benefit from a preference for SCV when appropriate to limit the risk of intravascular complications associated with prolonged central venous catheterization.

Despite its potential important consequences, only one previous study investigated the possibility of predicting catheter dwell time [11]. The authors found that clinicians had poor ability to predict the catheter dwell time and raised awareness towards the need to identify factors associated with an expected long catheter dwell time. Using a multivariable logistic regression model in which early deceased patients had short catheter duration, the authors found that MV was associated with longer catheter dwell time and that cancer was associated with shorter catheter dwell time. In our study, the use of Fine and Gray models allowed to account for death as a competing event, as recommended [16, 23]. Immunosuppression, defined as a composite of either solid or hematological malignancy or HIV infection, was associated with longer catheter dwell time. In those patients, the severity of the underlying disease may explain longer length of stay and therefore longer catheter dwell times [24]. Of note, strategies to prevent catheter-related infectious complications are particularly important in these patients since they are particularly exposed to intravascular catheters [9, 25]. Moreover, in addition to being more prone to develop infectious complications, such complications are more severe in immunocompromised patients [26].

Since MV did not verify the PH assumption [15, 17], it could not be entered as an explainable covariate in the analyses. Nevertheless, as suggested by *Holmberg *et al. [11], catheter dwell time is likely to be longer in ventilated patients who tend to be more severe, to have more organs dysfunctions, and who need continuous drugs infusions.

In contrast with *Holmberg *et al., we found an association between vasopressors use and longer catheter dwell time. Once again, this might be explained by the methodology and the severity of the underlying disease, reflecting organ dysfunction and expected longer ICU stay. Of note, vasopressors infusion through a catheter may facilitate biofilm formation, and then facilitate colonization [27, 28]. Moreover, a possible catecholamine-induced innate-immunity suppression has previously been described [29, 30].

We also found an association between obesity and longer catheter dwell time. Interestingly, this factor has been described in the literature as a risk factor for catheter colonization and CRI [31, 32], as well as for DVT [33, 34]. Strategies to prevent catheter-related intravascular complications could thus be particularly beneficial in these patients.

Of note, several factors identified in our study as risk factors for longer dwell time have also been described as risk factors for catheter-related intravascular complications, which somewhat increases the external validity of the CVC-IN score. Identifying patients with longer expected catheter dwell time could then allow to adopt strategies to prevent catheter-related intravascular complications upon catheter insertion, such as avoiding femoral insertion, favoring subclavian insertion in the absence of contra-indication.

In addition, the use of CVC-IN may also be useful in the choice of the CVA. The Michigan Appropriateness Guide for Intravenous Catheters guideline recommends PICC lines for ICU patients requiring at least 14 days of CVA [35]. Noteworthy, a review of current practices revealed that the main reason for non-adherence with these guidelines was the use of PICC lines for less than 14 days [35, 36].

This study has some limitations. First, the primary endpoint, namely required catheter dwell time was not directly measured in the trials. Instead, we used the prospectively collected reason for catheter removal to retrospectively proxy this endpoint. Therefore, our results rely on the accuracy of the declared reason and the way we summarized this endpoint for each patient. However, even though guidelines recommend removing nonessential catheters as quickly as possible [8], the definition of the lack of further utility is not standardized, and remains at the discretion of the healthcare workers in charge of each patient. Reassuringly, the several sensitivity analyses conducted were consistent. Second, we studied ventilated adult patients in the ICU. Therefore, our results may not be generalizable to unventilated patients, children or outside the ICU setting. Third, although data collection was prospective in all three RCTs, unmeasured covariates or missing data may cause residual confounding. The analysis was restricted to variables available in the original databases, then some potential risk factors may have been missed and not included in the CVC-IN score. Fourth, we did not include patients with PICC lines. However, the need for central venous access is associated with patients’ condition and independent of the type central venous access. Therefore, we think our results are generalizable to centers using PICC lines. Fifth, the ability of CVC-IN score to separate patients at the time of CVC insertion between expected shorter and longer CVC dwell time although significant, was modest. Nevertheless, the consequence of being wrong with CVC-IN is also modest: not supporting a subclavian site for a patient needing longer CVC dwell time. Of note, the current strategy in most ICUs worldwide is to prefer ultrasound-guided internal jugular catheterization for all. In addition, we restricted the CVC-IN score to variables available at the time of ICU admission, therefore disqualifying SAPS-2 score, which increased the discrimination of this score (data not shown). Finally, the three multicenter RCTs were conducted in France, which potentially limits the results generalizability.

Our results might have implications for clinical research. Future randomized, controlled trials investigating the prevention of CVC-related intravascular complications may gain additional internal validity by stratifying randomization based on the CVC-IN score, to increase arm comparability regarding the catheter-days. Moreover, the duality between the immediate risk of mechanical complications during CVC insertion and the delayed risk of intravascular complications such as CRI or DVT calls for more research assessing the real morbidity associated with these events. For clinical practice, use of the CVC-IN score, based on clinical or biological variables available on ICU admission, may help intensivists select the best anatomic site for catheter insertion, with an optimal balance between mechanical complications and the infectious or thrombotic risks according to the predicted need for CVA. The CVC-IN score might be improved in the future, by including dynamic variables for instance.

## Conclusions

In conclusion, the CVC-IN score showed a modest ability to discriminate which ventilated patients were expected to have longer required catheter dwell time. Whether the use of CVC-IN is clinically effective to optimize the strategy for catheter-related intravascular complications prevention requires further prospective evaluation.

## Supplementary Information

**Additional file 1: Annex 1.** Detailed presentation of the randomized controlled trials included in the study. **Annex 2.** Detailed statistical method. **Annex 3.** Method for the determination of the attached points in the points-based system (1). **Figure S1.** Calculation of the required catheter dwell time **Figure S2.** Schoenfeld residuals for covariates included in the analyses **Figure S3.** Cumulative Incidence of catheter removal for absence of further utility and death in the training cohort (n=2336). **Figure S4.** Cumulative Incidence of catheter removal for absence of further utility and death in the testing cohort (n=2371**). Figure S5.** Cumulative Incidence of catheter removal for absence of by points total of the CVC-IN score in the overall cohort (n=4707). **Figure S6.** Cumulative Incidence of catheter removal for absence of further utility and death in the overall cohort (n=4707). **Table S1.** Outcomes in the training and testing cohorts. **Table S2.** Variance Inflation Factors for the covariates included in the multivariable analysis. **Table S3.** Univariable and multivariable subdistribution hazard models for catheter removal in the five time imputed dataset. **Table S4.** Robust risk factors associated with absence of further utility after 500 bootstrap. **Table S5.** Univariable and multivariable subdistribution hazard models for catheter removal in the training cohort considering only the first catheter in ventilated patients (n=2336). **Table S6.** Univariable and multivariable subdistribution hazard models for catheter removal in the training cohort considering the catheters inserted > 72 hours after removal of the previous catheter (n=2336).

## Data Availability

The datasets used during the current study are available from the corresponding author on reasonable request.

## Abbreviations

AUC: Area under curve
BMI: Body mass index
CI: Confidence interval
CRI: Catheter related infection
CVA: Central venous access
CVC: Central venous catheter
DVT: Deep vein thrombosis
HIV: Human immunodeficiency virus
HR: Hazard ratio
ICU: Intensive care unit
IJV: Internal jugular vein
IQR: Interquartile range
MV: Mechanical ventilation
NPV/PPV: Negative predictive value/positive predictive value
PH: Proportional hazard
RCT: Randomized controlled trial
ROC: Receiving operator characteristics
SAPS: Simplified Acute Physiology Score
SCV: Subclavian vein
