# Supplementary material for: Development and validation of a multivariable model predicting the required catheter dwell time among mechanically ventilated critically ill patients in three randomized trials
Source: Ann Intensive Care. 2023 Jan 16;13:5. doi: 10.1186/s13613-023-01099-9 (PMC9842826; doi:10.1186/s13613-023-01099-9)
Supplement: Supplementary file 1 — Additional file 1: Annex 1. Detailed presentation of the randomized controlled trials included in the study. Annex 2. Detailed statistical method. Annex 3. Method for the determination of the attached points in the points-based system (1). Figure S1. Calculation of the required catheter dwell time Figure S2. Schoenfeld residuals for covariates included in the analyses Figure S3. Cumulative Incidence of catheter removal for absence of further utility and death in the training cohort (n=2336). Figure S4. Cumulative Incidence of catheter removal for absence of further utility and death in the testing cohort (n=2371). Figure S5. Cumulative Incidence of catheter removal for absence of by points total of the CVC-IN score in the overall cohort (n=4707). Figure S6. Cumulative Incidence of catheter removal for absence of further utility and death in the overall cohort (n=4707). Table S1. Outcomes in the training and testing cohorts. Table S2. Variance Inflation Factors for the covariates included in the multivariable analysis. Table S3. Univariable and multivariable subdistribution hazard models for catheter removal in the five time imputed dataset. Table S4. Robust risk factors associated with absence of further utility after 500 bootstrap. Table S5. Univariable and multivariable subdistribution hazard models for catheter removal in the training cohort considering only the first catheter in ventilated patients (n=2336). Table S6. Univariable and multivariable subdistribution hazard models for catheter removal in the training cohort considering the catheters inserted > 72 hours after removal of the previous catheter (n=2336). [file 13613_2023_1099_MOESM1_ESM.docx]

Additional files

Development and validation of a multivariable model predicting the required catheter dwell time amongst ventilated critically ill patients in three randomized trials

Jeanne Iachkine, MS¹⁻²; Niccolò Buetti, MD, MSc³; Harm-Jan de Grooth, MD, PhD⁴; Anaïs R Briant, MS⁵; Olivier Mimoz, MD, PhD ⁶; Bruno Mégarbane, MD, PhD ⁷; Jean-Paul Mira, MD, PhD ⁸; Xavier Valette, MD⁹; Cédric Daubin, MD⁹; Damien du Cheyron, MD, PhD ⁹; Leonard A Mermel, MD, PhD ¹⁰⁻¹¹; Jean-François Timsit, MD, PhD ¹²; Jean-Jacques Parienti, MD, PhD¹⁻²

¹ Department of Clinical Research and Biostatistics, Caen University Hospital and Caen Normandy University, Caen, France.

² INSERM U1311 DYNAMICURE, Caen Normandy University, Caen, France.

³ Infection Control Program and World Health Organization Collaborating Center on Patient Safety, Hospitals and Faculty of Medicine, University of Geneva, Geneva, Switzerland

⁴ Department of Intensive Care, Amsterdam UMC location Vrije Universiteit Amsterdam, De Boelelaan 1117, Amsterdam, The Netherlands

⁵ Department of Biostatistics and Clinical Research, Caen University Hospital, Avenue de la Côte de Nacre, CS 30001, F-14000 Caen, France.

⁶ Inserm U1070, Poitiers University, Poitiers, France; Poitiers University Hospital, Poitiers, 86021, France.

⁷ Medical and Toxicological Intensive Care Unit, Lariboisière Hospital, AP-HP, INSERM UMRS-1144, Paris University, Paris, France.

⁸ Medical ICU, Cochin Hospital, AP-HP, 27 rue du Faubourg Saint-Jacques, 75014, Paris, France.

⁹ Department of Medical Intensive Care, Caen University Hospital, 14000, Caen, France.

¹⁰ Department of Epidemiology and Infection Prevention, Lifespan Hospital System, Providence, Rhode Island, USA.

¹¹ Department of Medicine, Warren Alpert Medical School of Brown University, Providence, Rhode Island, USA.

¹² Medical and infectious diseases ICU (MI2), Bichat Hospital, AP-HP, University of Paris, IAME, INSERM U1137, Paris, France.

Correspondance to: Pr. Jean-Jacques Parienti, [parienti-jj@chu-caen.fr](mailto:parienti-jj@chu-caen.fr)

Table of contents

[**Annex 1:** Detailed presentation of the randomized controlled trials included in the study. 3](#_Toc120615562)

[**Additional file 1: Figure S1:** Calculation of the required catheter dwell time. 6](#_Toc120615563)

[**Annex 2:** Detailed statistical method 7](#_Toc120615564)

[**Additional file 1: Figure S2:** Schoenfeld residuals for covariates included in the analyses. 12](#_Toc120615565)

[**Annex 3:** Method for the determination of the attached points in the points-based system (1) 13](#_Toc120615566)

[**Additional file 1: Table S1:** Outcomes in the training and testing cohorts 16](#_Toc120615567)

[**Additional file 1: Table S2:** Variance Inflation Factors for the covariates included in the multivariable analysis 16](#_Toc120615568)

[**Additional file 1: Table S3:** Univariable and multivariable subdistribution hazard models for catheter removal in the five time imputed dataset 17](#_Toc120615569)

[**Additional file 1: Table S4:** Robust risk factors associated with absence of further utility after 500 bootstrap 17](#_Toc120615570)

[**Additional file: Figure S3:** Cumulative Incidence of catheter removal for absence of further utility and death in the training cohort (n=2,336) 18](#_Toc120615571)

[**Additional file 1: Figure S4:** Cumulative Incidence of catheter removal for absence of further utility and death in the testing cohort (n=2,371) 19](#_Toc120615572)

[**Additional file 1: Figure S5:** Cumulative Incidence of catheter removal for absence of by points total of the CVC-IN score in the overall cohort (n=4,707) 20](#_Toc120615573)

[**Additional file 1: Figure S6**: Cumulative Incidence of catheter removal for absence of further utility and death in the overall cohort (n=4,707) 21](#_Toc120615574)

[**Additional file 1: Table S5:** Univariable and multivariable subdistribution hazard models for catheter removal in the training cohort considering only the first catheter in ventilated patients (n=2,336) 22](#_Toc120615575)

[**Additional file 1: Table S6:** Univariable and multivariable subdistribution hazard models for catheter removal in the training cohort considering the catheters inserted > 72 hours after removal of the previous catheter (n=2,336) 23](#_Toc120615576)

[**Additional file 1: Table S7:** Univariable and multivariable Cox cause-specific models for catheter removal for absence of further utility and death in the training cohort (n=2,336) 24](#_Toc120615577)

# **Annex 1:** Detailed presentation of the randomized controlled trials included in the study.

**A. Training cohort**

The 3SITES (1) study compared subclavian, jugular and femoral access towards major central catheter related complication, defined as the composite of catheter related blood stream infection and symptomatic deep vein thrombosis.

Recruitment was conducted from December 2011 to June 2014. Patients were recruited from 10 French ICUs, in four university-affiliated hospitals and five general hospitals and were followed until death or Intensive Care Unit (ICU) discharge.

Inclusion criteria were adult patients (≥ 18 years) admitted to ICU, requiring catheterization with a central venous catheter through a new venipuncture, and considered to be suitable for insertion in at least two sites (among internal jugular veins, subclavian veins and femoral veins).

Patients were randomized in a 1:1:1 randomization scheme if all the three venous sites were suitable for catheter placement. If one of the three sites was not suitable on both the left and right sides of the body, the catheterization site was assigned in a 1:1 randomization scheme for the other two sites (two-choice scheme). If only one site was suitable, the catheterization procedure was not included in the study. Randomization was stratified according to

ICU and according to the use of antibiotic therapy.

The primary outcome was the incidence of major catheter related complications from the time of insertion to 48 hours after catheter removal. Major complications were defined as the composite of catheter-related bloodstream infection (CRBSI) and symptomatic deep-vein thrombosis, whichever occurred first.

**B. Testing cohort**

The CLEAN (2) study compared skin disinfection using chlorhexidine-alcohol versus povidone iodine alcohol regarding the incidence of catheter-related infection.

Recruitment was conducted between October 2012 and February 2014, in 11 French ICUs in

5university-affiliated hospitals and 1 general hospital. Patients were followed until death or 48 hours after ICU discharge. Eligible patients were consecutive critically ill adult patients requiring arterial, dialysis or central venous catheters for at least 48 hours. Exclusion criteria were contraindication to any trial antiseptic, high risk of death within the 48 first hours or need to use an antimicrobial-coated catheter. Patients were randomly assigned in a 1:1:1:1 ratio to one of the four treatment groups (chlorhexidine alcohol or povidone iodine alcohol, with administration preceded or not by skin scrubbing with an antiseptic detergent).

The primary outcome was the incidence of CRI.

Finally, the DRESSING2 (3) study compared three types of transparent dressings regarding the catheter colonization rate and the major catheter related infection rate.

Recruitment was conducted from May 2010 to July 2011, in 12 ICUs in 7 university-affiliated hospitals and 4 general hospitals. Patients were followed until death or 48 hours after ICU discharge. Eligible patients were consecutive ICU patients expected to require intravascular catheterization for at least 48 hours. Exclusion criterion was known allergy to chlorhexidine or to transparent dressings. Patients were randomly assigned to one of the three dressings (chlorhexidine gel impregnated dressing or highly adhesive dressing or standard transparent dressing). Randomization was stratified by ICU. The primary outcome was either the catheter colonization rate for highly adhesive dressings versus standard dressings or major CRI rate for chlorhexidine impregnated dressings versus non chlorhexidine dressings. Major CRI was defined as CRBSI or catheter-related sepsis without CRBSI.

**C. Trials Procedures**

Insertion took place in the ICU, and was performed by junior or senior physicians depending on the studies. Maximal sterile barrier precautions including surgical hand antisepsis, use of large sterile drapes, and use of sterile gowns and gloves were used. Skin disinfection used either alcoholic povidone iodine or alcoholic chlorhexidine.

Catheterization was achieved by means of the Seldinger technique, with the use of anatomical landmarks or ultrasonographic guidance. Catheters were not used for routine blood sampling. Catheters were removed when no longer required, or when a new access was required, as decided independently by the physicians caring for each patient. Patients discharged from the ICU with the catheter in place had blood cultures drawn from the catheter and from a peripheral vein simultaneously to take account of the absence of catheter-tip culture.

**References:**

1. Parienti J-J, Mongardon N, Mégarbane B, Mira J-P, Kalfon P, Gros A, Marqué S, Thuong M, Pottier V, Ramakers M, Savary B, Seguin A, Valette X, Terzi N, Sauneuf B, Cattoir V, Mermel LA, du Cheyron D (2015) Intravascular Complications of Central Venous Catheterization by Insertion Site. New England Journal of Medicine 373(13):1220-1229. <https://www.nejm.org/doi/10.1056/NEJMoa1500964>
2. Mimoz O, Lucet J-C, Kerforne T, Pascal J, Souweine B, Goudet V, Mercat A, Bouadma L, Lasocki S, Alfandari S, Friggeri A, Wallet F, Allou N, Ruckly S, Balayn D, Lepape A, Timsit J-F (2015) Skin antisepsis with chlorhexidine–alcohol versus povidone iodine–alcohol, with and without skin scrubbing, for prevention of intravascular-catheter-related infection (CLEAN): an open-label, multicentre, randomised, controlled, two-by two factorial trial. The Lancet 386:2069–2077. <https://doi.org/10.1016/S0140-6736(15)00244-5>
3. Timsit J-F, Mimoz O, Mourvillier B, Souweine B, Garrouste-Orgeas M, Alfandari S, Plantefeve G, Bronchard R, Troche G, Gauzit R, Antona M, Canet E, Bohe J, Lepape A, Vesin A, Arrault X, Schwebel C, Adrie C, Zahar, J-R, Ruckly S, Trounegros C, Lucet J-C (2012) Randomized Controlled Trial of Chlorhexidine Dressing and Highly Adhesive Dressing for Preventing Catheter-related Infections in Critically Ill Adults. American Journal of Respiratory and Critical Care Medicine 186(12):1272-1278. ttps://doi.org/10.1164/rccm.201206-1038OC

#
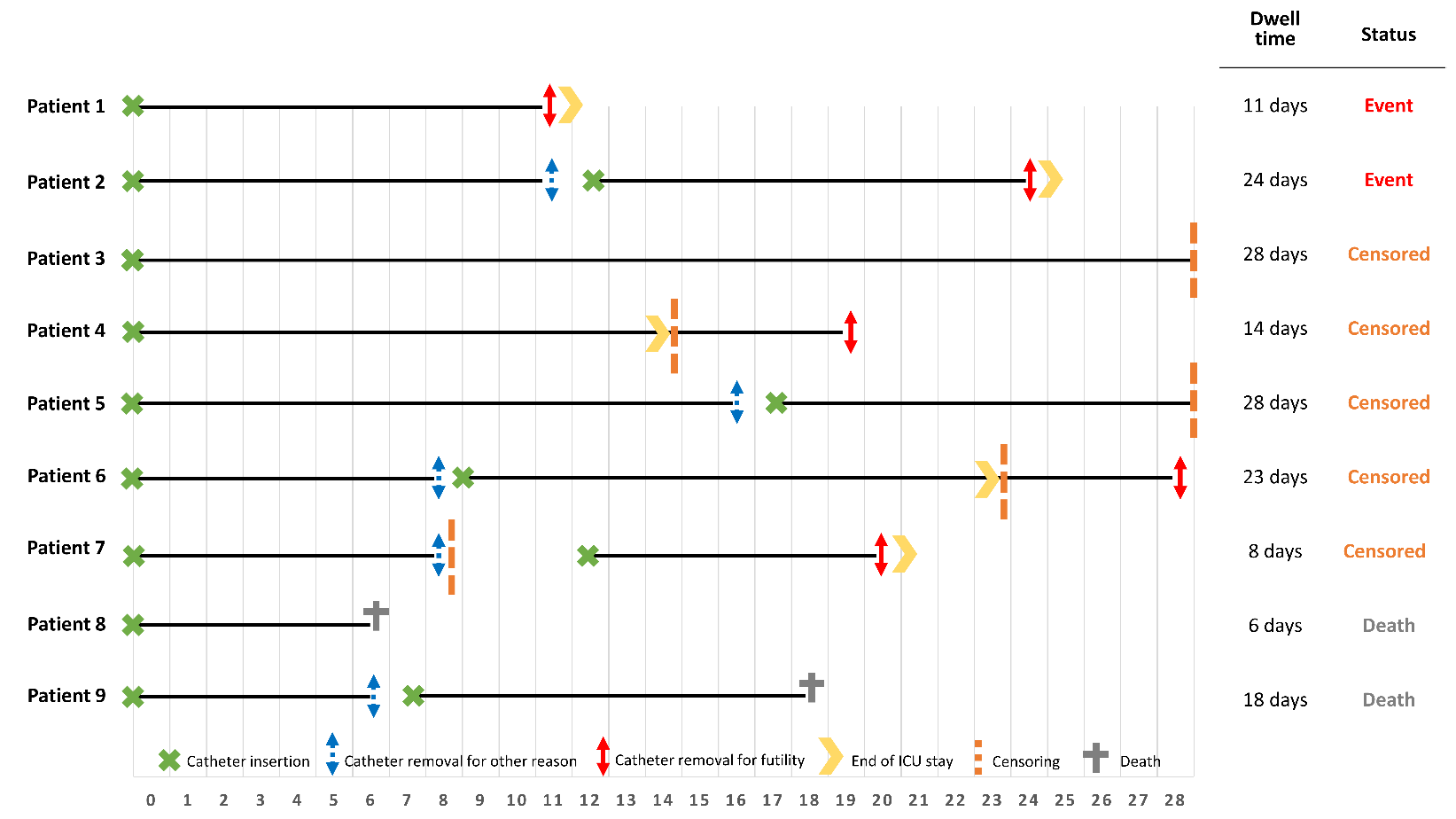
**Additional file 1: Figure S1:** Calculation of the required catheter dwell time.

#

# **Annex 2:** Detailed statistical method

No computation of sample size was performed *a priori*. Baseline characteristics of the patients and catheters included in the study were described using numbers (percentages) for categorical variables, and median [inter-quartile range] for quantitative variables.

Demographic data such as age and sex category were recorded at admission. The Simplified Acute Physiology Score (SAPS2) was calculated within the first 24 hours following ICU admission and was used to assess the severity of disease. Past medical history, especially immunosuppression (defined as a composite of Human Immunodeficiency Virus infection, and solid or hematological malignancies), obesity, diabetes, was recorded for every patient. Data concerning medication at the time of catheterization was recorded regarding antibiotic therapy, anticoagulation with unfractionated heparin and vasopressors in the three RCTs. Dates of insertion and removal were used to calculate the indwell time for each catheter. For patients in whom several catheters were inserted during the stay, the total catheter indwell time was calculated by summing the respective indwell times for each successive catheter until removal for absence of further utility, death or censoring.

Body Mass Index (BMI) was categorized as ≥ 30 kg/m² defining obesity according to the World Health Organization (WHO) definition. Age was categorized in six categories to match the categories used for the Simplified Acute Physiology Score II calculation. Creatinine level at admission was dichotomized as < or ≥ 100 µmol/L (median value in the training cohort). Time to catheter removal for absence of further utility was used as a continuous time-dependant variable.

Assuming a missing at random pattern, single imputation of missing data regarding key independent variables was performed to allow exploitation of all observations. BMI was imputed as predicted by linear regression of age and sex. For qualitative variables, a missing data was considered as the absence of the event.

First, we investigated potential risk factors associated with total catheter indwell time. Baseline variables considered as clinically relevant were assessed regarding the proportional hazard assumption using the graphical representation of Schoenfeld residuals (1). This assumption was not verified for mechanical ventilation, use of antibiotics and use of unfractionated heparin. Therefore, analyses were conducted in the subgroup of patients requiring mechanical ventilation, and the two latter variables (antibiotics and unfractionated heparin use) were not included in the models. We used a subdistribution hazard model, also called Fine and Gray model to take account for the competing risk of death (2,3).

All variables associated with total catheter indwell time (p-value <0.20) in univariate analysis were selected for inclusion in the multivariate analysis. We assessed multicolinearity between these variables by calculating the variance inflation factor (VIF). The VIF did not exceed 5 for any variable, denoting the absence of collinearity. We used backward and forward selection procedures among qualified variables to select independent risk factors associated with time to catheter removal for uselessness and we verified the absence of second-order interactions. A threshold of p <0.05 to remain in or enter the model was chosen to decrease the number of variables finally included in the predictive score, to make it easier and more practical to use. At this stage, the same univariable and multivariable models were conducted in a five time imputed dataset in order to ensure that the same risk factors were identified with both imputation strategies.

Second, we assessed the internal validity (stability and consistency) of our model, by conducting the same multivariate Fine and Grays subdistribution hazard model in 500 bootstrap samples drawn from the training cohort, which allowed to assess the distribution of an indicator variable specifying the statistical significance (p < 0.05) for each predictor variable. This procedure was performed in the full-sized training cohort, and in random subsamples containing respectively 90%, 80% and 70% of the full-sized training cohort. We considered a risk factor robust when found significant in at least 50 % of the bootstrap samples and subsamples, as described by *Mannan* (4). Subdistribution hazards-ratios (HR) and coefficients for the robust risk factors also were assessed using bootstrap samples.

The CVC-IN score was then computed using robust risk factors and their bootstrapped coefficients. The score was developed as a simple points-based system, using the method described by *Austin et al*. (5). The constant for the points-based system (number of regression units corresponding to one point) was defined as the increase of risk associated with vasopressors use. Predictors states associated with longer time to catheter removal for absence of further utility were assigned more points, meaning a higher point total represents a longer total catheter indwell time. The predictive value of the CVC-IN score was tested in the training cohort using the area under 28-days time dependent receiver operating characteristic (ROC) curves (AUC), to estimate the sensitivity, specificity, predictive positive value and negative predictive value. The CVC-IN score was dichotomized as “low” and “high” categories, using its median value in the training cohort as threshold. Cumulative incidence functions (CIF) curves were drawn for the dichotomized CVC-in score in the training cohort

To compute the CVC-IN score in the testing cohort in the absence of recorded data for the baseline creatinine, the covariate “Creatinine > 100 µmol/L” was imputed. Patients in whom a dialysis catheter was inserted at ICU admission were considered as having creatinine levels > 100 µmol/L. For other patients, a propensity score was built to assess the probability of having creatinine levels > 100 µmol/L, based on age, gender, BMI, diabetes, vasopressors use, immunosuppression and antibiotics use. Patients with a propensity -score predicted probability > 0.5 were considered as having creatinine levels > 100 µmol/L.

The predictive value of the CVC-IN score was assessed in the testing cohort using time dependent ROC-curves and their AUC and its calibration was assessed using a calibration plot and computation of the Brier score (6). Cumulative incidence functions (CIF) curves were drawn for the dichotomized CVC-in score in the testing cohort and in the overall cohort, constituted by pooling the training and the testing cohorts.

We performed several sensitivity analyses. The first one consisted in conducting univariable and multivariable Fine and Gray subdistribution hazard models in the training cohort, by taking into account only the first catheter of each patient. The second one consisted in conducting univariable and multivariable Fine and Gray subdistribution hazard models in the training cohort, taking into account all the catheters inserted until day 28, regardless the time between catheter removal and reinsertion of a new catheter. Finally, we conducted univariable and multivariable Cox models for death and catheter removal for absence of further utility, to obtain cause specific hazard ratios for both events. All analyses were performed with SAS software V9.4 (SAS Institute, NC, Cary), and R software (R Foundation for Statistical Computing, Vienna, Austria), packages cmprsk, riskRegression and timeROC.

**References:**

1. Schoenfeld D. Partial residuals for the proportional hazards regression model. Biometrika. 1982;69(1):239–41.

2. Fine JP, Gray RJ. A Proportional Hazards Model for the Subdistribution of a Competing Risk. J Am Stat Assoc. 1999 Jun;94(446):496–509.

3. Brock GN, Barnes C, Ramirez JA, Myers J. How to handle mortality when investigating length of hospital stay and time to clinical stability. BMC Med Res Methodol. 2011 Dec;11(1):144.

4. Mannan H. A practical application of a simple bootstrapping method for assessing predictors selected for epidemiologic risk models using automated variable selection. Int J Stat Appl. 2017 Sep 1;7:239–49.

5. Austin PC, Lee DS, D’Agostino RB, Fine JP. Developing points-based risk-scoring systems in the presence of competing risks: Competing Risks and Risk Scores. Stat Med. 2016 Sep 30;35(22):4056–72.

6. Gerds TA, Andersen PK, Kattan MW. Calibration plots for risk prediction models in the presence of competing risks. Stat Med. 2014 Aug 15;33(18):3191–203.

#
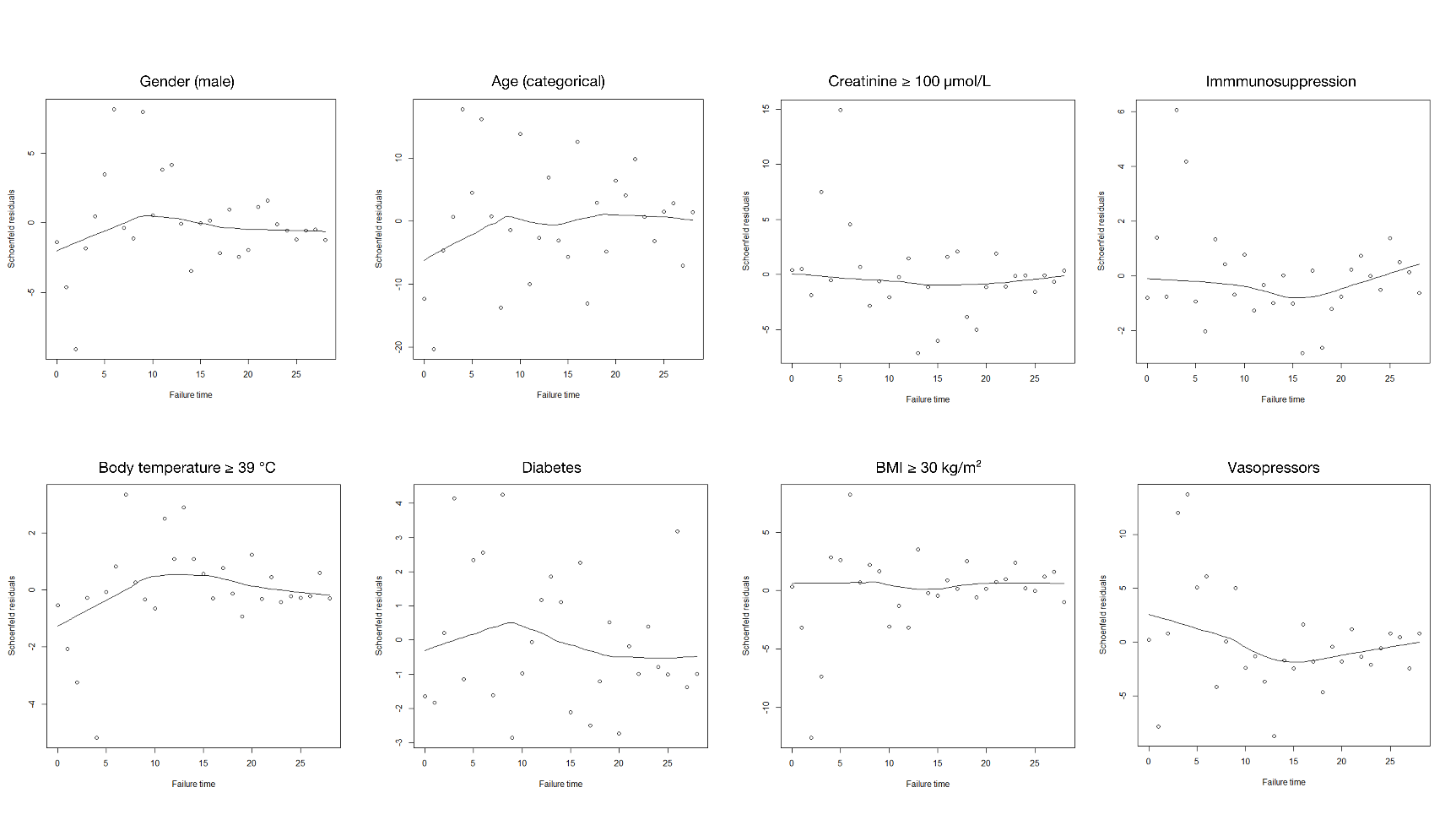
**Additional file 2: Figure S2**: Schoenfeld residuals for covariates included in the analyses.

# **Annex 3**: Method for the determination of the attached points in the points-based system (1)

**Step 1: Obtain multivariate regression coefficients**

| **Risk factors** | **Bootstrapped coefficients (βi)** |
| --- | --- |
| **Age (years)*** |  |
| **40-59** | -0.36 |
| **60-69** | -0.48 |
| **70-74** | -0.54 |
| **75-80** | -0.58 |
| **≥ 80** | -0.48 |
| **Obesity** | -0.16 |
| **Vasopressors** | -0.25 |
| **Immunosuppression** | -0.46 |
| **Creatinine > 100 µmol/L** | -0.42 |
| * Age is coded as a dummy variable. Reference category is “< 40 years”. | |

**Step 2: Definition of reference values, and referent risk profiles**

| **Risk factors** | **Categories** | **Reference value (Wij)** | **Referent risk profile** |
| --- | --- | --- | --- |
| **Age 40-59 years*** | 0 | 0.00 | 0.00 |
| **Age 40-59 years*** | 1 | 1.00 | 0.00 |
| **Age 60-69 years*** | 0 | 0.00 | 0.00 |
| **Age 60-69 years*** | 1 | 1.00 | 0.00 |
| **Age 70-74 years*** | 0 | 0.00 | 0.00 |
| **Age 70-74 years*** | 1 | 1.00 | 0.00 |
| **Age 75-80 years*** | 0 | 0.00 | 0.00 |
| **Age 75-80 years*** | 1 | 1.00 | 0.00 |
| **Age ≥ 80 years*** | 0 | 0.00 | 0.00 |
| **Age ≥ 80 years*** | 1 | 1.00 | 0.00 |
| **Obesity** | 0 | 0.00 | 0.00 |
| **Obesity** | 1 | 1.00 | 0.00 |
| **Vasopressors** | 0 | 0.00 | 0.00 |
| **Vasopressors** | 1 | 1.00 | 0.00 |
| **Immunosuppression** | 0 | 0.00 | 0.00 |
| **Immunosuppression** | 1 | 1.00 | 0.00 |
| **Creatinine > 100 µmol/L** | 0 | 0.00 | 0.00 |
| **Creatinine > 100 µmol/L** | 1 | 1.00 | 0.00 |
| * Age is coded as a dummy variable. Reference category is “< 40 years”. | | | |

|  |
| --- |

**Step 3**: **Establish distance from base category**

We now compute how far each category of each risk factor is from the base category in terms of regression unites using distance = $\beta_{i}(W_{ij}-W_{iREF})$

| **Risk factors** | **Categories** | **Reference value (Wij)** | **Referent risk profile** | **Bootstrapped coefficient (βi)** | **Distance from base category** |
| --- | --- | --- | --- | --- | --- |
| **Age 40-59 years*** | 0 | 0.00 | 0.00 | -0.36 | 0.00 |
| **Age 40-59 years*** | 1 | 1.00 | 0.00 | -0.36 | 0.36 |
| **Age 60-69 years*** | 0 | 0.00 | 0.00 | -0.48 | 0.00 |
| **Age 60-69 years*** | 1 | 1.00 | 0.00 | -0.48 | 0.48 |
| **Age 70-74 years*** | 0 | 0.00 | 0.00 | -0.54 | 0.00 |
| **Age 70-74 years*** | 1 | 1.00 | 0.00 | -0.54 | 0.54 |
| **Age 75-80 years*** | 0 | 0.00 | 0.00 | -0.58 | 0.00 |
| **Age 75-80 years*** | 1 | 1.00 | 0.00 | -0.58 | 0.58 |
| **Age ≥ 80 years*** | 0 | 0.00 | 0.00 | -0.48 | 0.00 |
| **Age ≥ 80 years*** | 1 | 1.00 | 0.00 | -0.48 | 0.48 |
| **Obesity** | 0 | 0.00 | 0.00 | -0.16 | 0.00 |
| **Obesity** | 1 | 1.00 | 0.00 | -0.16 | 0.16 |
| **Vasopressors** | 0 | 0.00 | 0.00 | -0.25 | 0.00 |
| **Vasopressors** | 1 | 1.00 | 0.00 | -0.25 | 0.25 |
| **Immunosuppression** | 0 | 0.00 | 0.00 | -0.46 | 0.00 |
| **Immunosuppression** | 1 | 1.00 | 0.00 | -0.46 | 0.46 |
| **Creatinine > 100 µmol/L** | 0 | 0.00 | 0.00 | -0.42 | 0.00 |
| **Creatinine > 100 µmol/L** | 1 | 1.00 | 0.00 | -0.42 | 0.42 |
| * Age is coded as a dummy variable. Reference category is “< 40 years”. | | | | | |

**Step 4**: **Set the constant B**

We now define the constant for the points system, namely the number of regression units that will correspond to one point. Here, we let B reflect the increase of risk associated with the need for vasopressors (B=0.25)

**Step 6: Determination of the points associated with each category of the risk factors**

We now define the points associated with each category using ${Points}_{ij}= \frac{\beta_{i}(W_{ij}-W_{iREF})}{B}$

The result is rounded to the nearest integer to obtain the points.

| **Risk factors** | **Category** | **Reference value (Wij)** | **Referent risk profile** | **Bootstrapped coefficient (βi)** | **Distance from base category** | **Distance**  **/B** | **Points** |
| --- | --- | --- | --- | --- | --- | --- | --- |
| **Age 40-59 years*** | 0 | 0.00 | 0.00 | -0.36 | 0.00 | 0.00 | 0 |
| **Age 40-59 years*** | 1 | 1.00 | 0.00 | -0.36 | 0.36 | 1.44 | 1 |
| **Age 60-69 years*** | 0 | 0.00 | 0.00 | -0.48 | 0.00 | 0.00 | 0 |
| **Age 60-69 years*** | 1 | 1.00 | 0.00 | -0.48 | 0.48 | 1.92 | 2 |
| **Age 70-74 years*** | 0 | 0.00 | 0.00 | -0.54 | 0.00 | 0.00 | 0 |
| **Age 70-74 years*** | 1 | 1.00 | 0.00 | -0.54 | 0.54 | 2.16 | 2 |
| **Age 75-80 years*** | 0 | 0.00 | 0.00 | -0.58 | 0.00 | 0.00 | 0 |
| **Age 75-80 years*** | 1 | 1.00 | 0.00 | -0.58 | 0.58 | 2.32 | 2 |
| **Age ≥ 80 years*** | 0 | 0.00 | 0.00 | -0.48 | 0.00 | 0.00 | 0 |
| **Age ≥ 80 years*** | 1 | 1.00 | 0.00 | -0.48 | 0.48 | 1.92 | 2 |
| **Obesity** | 0 | 0.00 | 0.00 | -0.16 | 0.00 | 0.00 | 0 |
| **Obesity** | 1 | 1.00 | 0.00 | -0.16 | 0.16 | 0.64 | 1 |
| **Vasopressors** | 0 | 0.00 | 0.00 | -0.25 | 0.00 | 0.00 | 0 |
| **Vasopressors** | 1 | 1.00 | 0.00 | -0.25 | 0.25 | 1.00 | 1 |
| **Immunosuppression** | 0 | 0.00 | 0.00 | -0.46 | 0.00 | 0.00 | 0 |
| **Immunosuppression** | 1 | 1.00 | 0.00 | -0.46 | 0.46 | 1.84 | 2 |
| **Creatinine > 100 µmol/L** | 0 | 0.00 | 0.00 | -0.42 | 0.00 | 0.00 | 0 |
| **Creatinine > 100 µmol/L** | 1 | 1.00 | 0.00 | -0.42 | 0.42 | 1.68 | 2 |
| * Age is coded as a dummy variable. Reference category is “< 40 years”. | | | | | | | |

**Reference**:

1. Austin PC, Lee DS, D’Agostino RB, Fine JP. Developing points-based risk-scoring systems in the presence of competing risks: Competing Risks and Risk Scores. Stat Med. 2016 Sep 30;35(22):4056–72.

# **Additional file 1: Table S1:** Outcomes in the training and testing cohorts

|  | **Training cohort (n=2,336)** | **Testing cohort (n=2,371)** |
| --- | --- | --- |
| **Catheters per patient,** n (%) |  |  |
| 1 | 2,059 (88.1) | 1,989 (83.9) |
| 2 | 213 (9.1) | 286 (12.1) |
| 3 | 45 (1.9) | 67 (2.8) |
| 4 | 15 (0.6) | 22 (0.9) |
| 5 | 4 (0.2) | 4 (0.2) |
| > 5 | 0 | 3 (0.1) |
| **Status at day 28,** n (%) |  |  |
| Censored | 446 (19.1) | 404 (17.0) |
| Catheter-free | 1,309 (56.0) | 1,257 (53.0) |
| Deceased | 581 (24.9) | 710 (30.0) |
| **Patients discharged with catheter in place,** n (%) | 82 (3.5) | 56 (2.4) |
| **Patients with total catheter dwell time > 28 days,** n (%) | 47 (2.0) | 147 (6.2) |
| **Patients with two catheters separated by > 72 hours,** n (%) | 77 (3.3) | 55 (2.3) |

# **Additional file 1: Table S2:** Variance Inflation Factors for the covariates included in the multivariable analysis

| **Risk factors** | **Variance Inflation Factor** |
| --- | --- |
| **Male** | 1.03 |
| **Age** | 1.08 |
| **Diabetes** | 1.06 |
| **Obesity** | 1.05 |
| **Vasopressors** | 1.05 |
| **Immunosuppression** | 1.01 |
| **Creatinine > 100 µM** | 1.11 |

# **Additional file 1: Table S3:** Univariable and multivariable subdistribution hazard models for catheter removal in the five time imputed dataset

|  | **Univariable analysis** | | | **Multivariable analysis** | | |
| --- | --- | --- | --- | --- | --- | --- |
| **Risk factors** | **HR¹** | **95 % CI²** | **p-value** | **Adjusted HR¹** | **95 % CI²** | **p-value** |
| **Male** | 0.87 | [0.79-0.97] | **0.014** | - | - | **-** |
| **Age (years)** |  |  | ***<0.001**** |  |  | ***<0.001**** |
| **< 40** | 1 | - | - | 1 | - | - |
| **40-59** | 0.65 | [0.54-0.78] | **<0.001** | 0.70 | [0.58-0.85] | **<0.001** |
| **60-69** | 0.54 | [0.44-0.66] | **<0.001** | 0.63 | [0.51-0.77] | **<0.001** |
| **70-74** | 0.49 | [0.39-0.62] | **<0.001** | 0.58 | [0.46-0.74] | **<0.001** |
| **75-80** | 0.45 | [0.36-0.57] | **<0.001** | 0.56 | [0.44-0.71] | **<0.001** |
| **≥ 80** | 0.51 | [0.41-0.63] | **<0.001** | 0.61 | [0.50-0.78] | **<0.001** |
| **Body temperature**  **≥ 39 °C** | 0.94 | [0.76-1.17] | 0.59 |  |  |  |
| **Diabetes** | 0.79 | [0.70-0.91] | **0.002** | - | - | **-** |
| **Obesity** | 0.81 | [0.71-0.92] | **0.002** | 0.82 | [0.72-0.94] | **0.002** |
| **Vasopressors** | 0.74 | [0.66-0.83] | **<0.001** | 0.83 | [0.74-0.94] | **0.002** |
| **Immunosuppression** | 0.62 | [0.51-0.75] | **<0.001** | 0.63 | [0.52-0.77] | **<0.001** |
| **Creatinine > 100 µM** | 0.59 | [0.53-0.66] | **<0.001** | 0.65 | [0.58-0.73] | **<0.001** |

** p-value is for type III effect,* ¹ HR, Hazard-Ratio, ² CI, Confidence Interval

# **Additional file 1: Table S4:** Robust risk factors associated with absence of further utility after 500 bootstrap

| **Risk factors** | **Full sample** | **90 % sample** | **80 % sample** | **70 % sample** | **Bootstrapped HR** | **Bootstrapped β** | **Points** |
| --- | --- | --- | --- | --- | --- | --- | --- |
| **Age** | **100** | **100** | **100** | **100** |  |  |  |
| **< 40 years** |  |  |  |  | 1 | 0 | 0 |
| **40-59 years** |  |  |  |  | 0.70 | -0.36 | 1 |
| **60-69 years** |  |  |  |  | 0.62 | -0.48 | 2 |
| **70-74 years** |  |  |  |  | 0.58 | -0.54 | 2 |
| **75-80 years** |  |  |  |  | 0.56 | -0.58 | 2 |
| **≥ 80 years** |  |  |  |  | 0.62 | -0.48 | 2 |
| **Obesity** | **72.2** | **67.4** | **56.4** | **70.2** | 0.85 | -0.16 | 1 |
| **Vasopressors** | **99.4** | **100** | **99.6** | **99.8** | 0.78 | -0.25 | 1 |
| **Immunosuppression** | **99.4** | **97.2** | **95.8** | **92.6** | 0.63 | -0.46 | 2 |
| **Creatinine > 100 µM** | **100** | **100** | **100** | **100** | 0.66 | -0.42 | 2 |

# **Additional file 1: Figure S3**: Cumulative Incidence of catheter removal for absence of further utility and death in the training cohort (n=2,336)


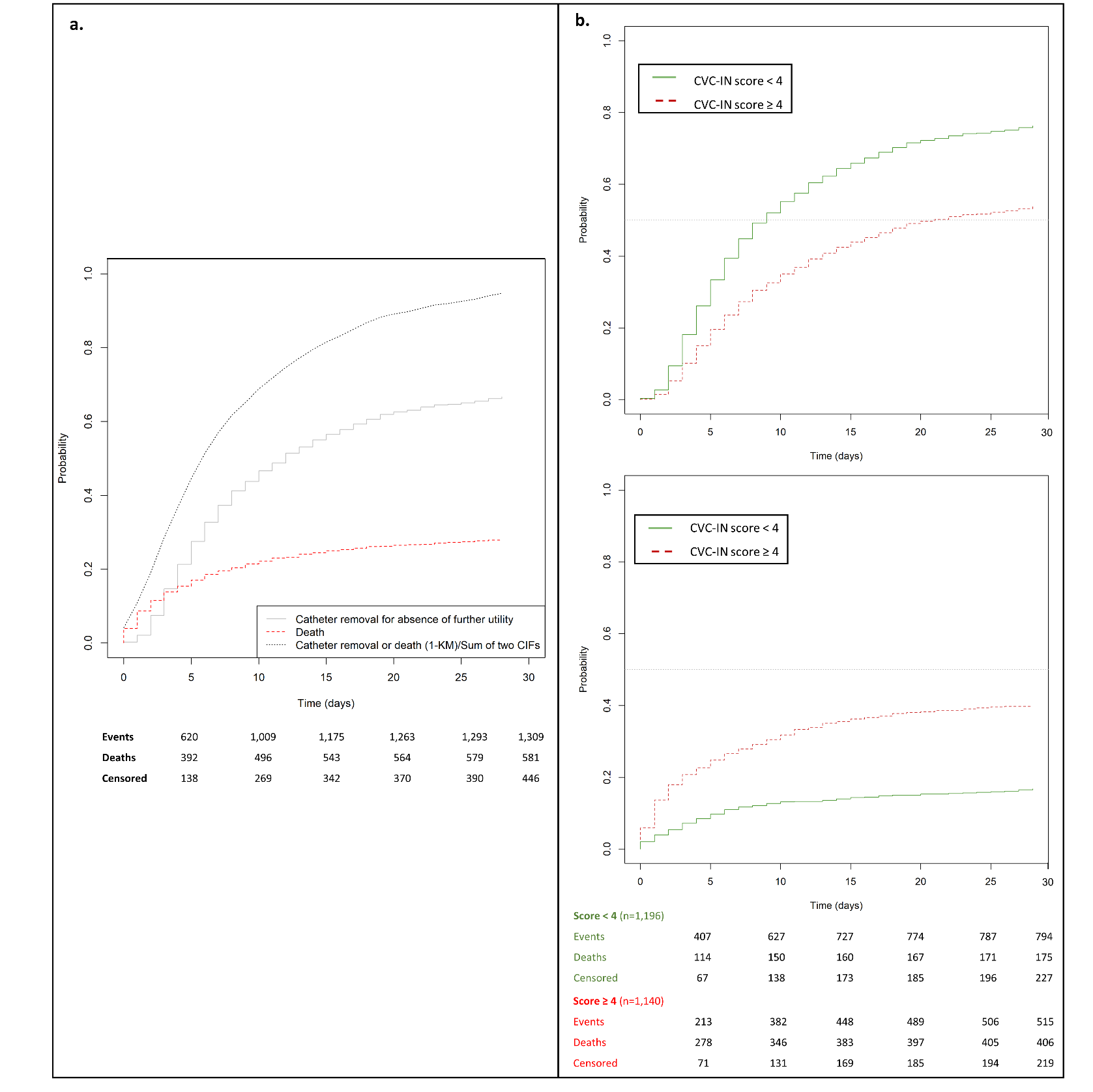


Panel (a.) displays the overall cumulative incidence of catheter removal and death in the training cohort. Panel (b.) displays the cumulative incidence of catheter removal (top figure) and death (bottom figure) according to the CVC-IN score.

# **Additional file 1: Figure S4**: Cumulative Incidence of catheter removal for absence of further utility and death in the testing cohort (n=2,371)


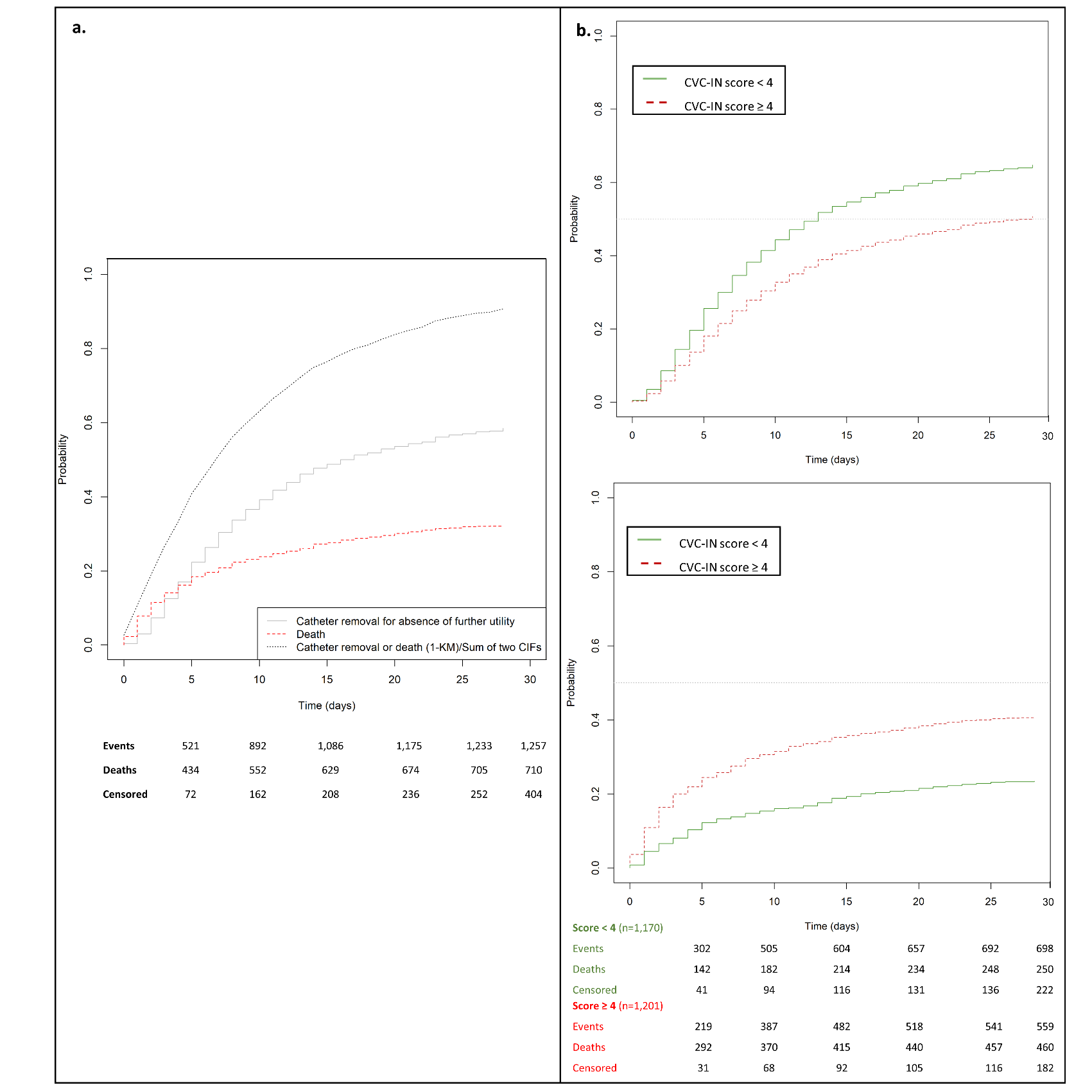


Panel (a.) displays the overall cumulative incidence of catheter removal and death in the testing cohort. Panel (b.) displays the cumulative incidence of catheter removal (top figure) and death (bottom figure) according to the CVC-IN score.

# **Additional file 1: Figure S5**: Cumulative Incidence of catheter removal for absence of by points total of the CVC-IN score in the overall cohort (n=4,707)


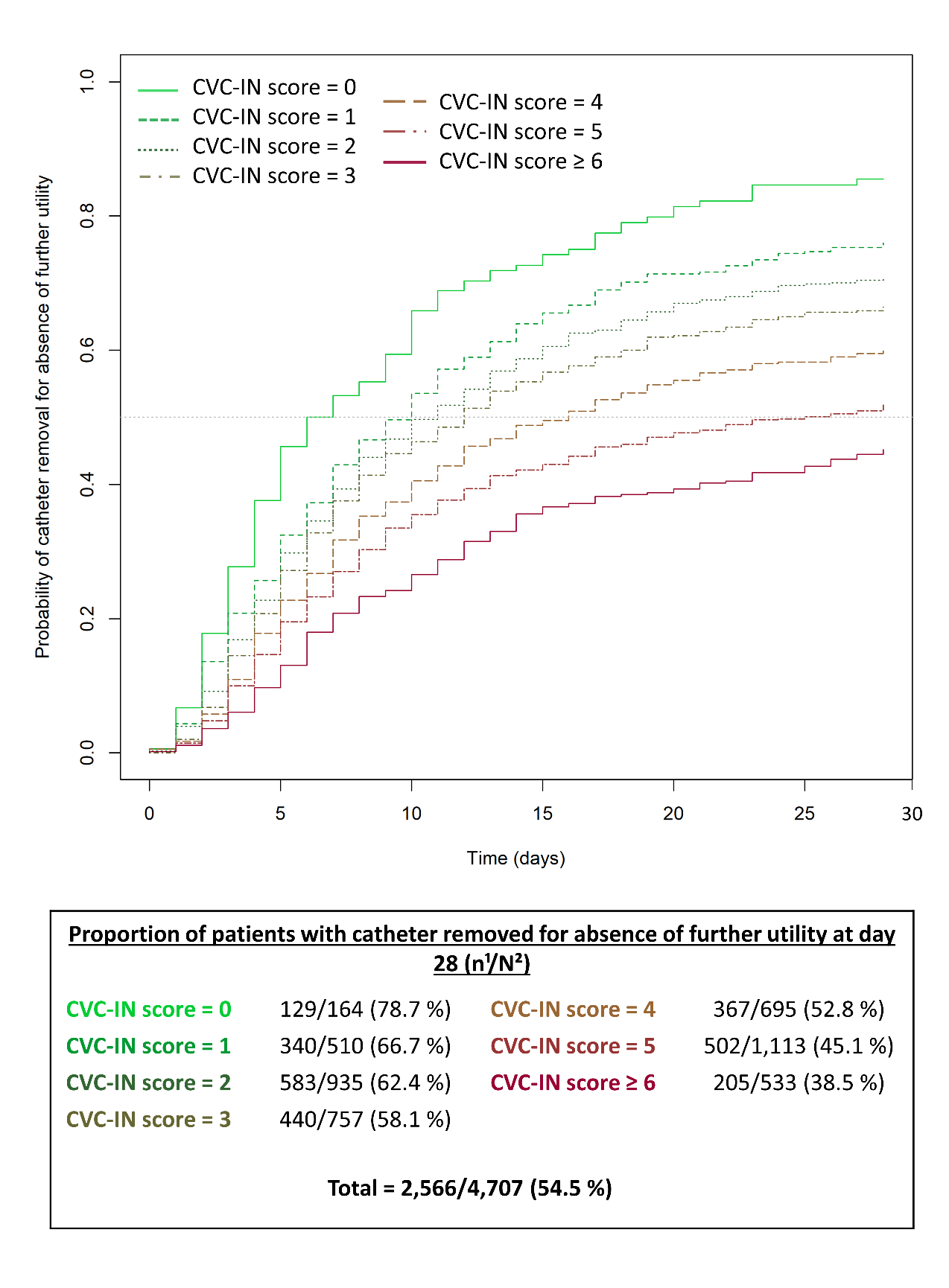


NB: points total 6, 7 and 8 were grouped since the number of patients in these categories were small.

¹ n is for number of patients in whom the catheter was removed for absence of further utility within day 28 by points total of the CVC-IN score; ² N is for the total number of patients by points total of the CVC-IN score.

# **Additional file 1: Figure S6**: Cumulative Incidence of catheter removal for absence of further utility and death in the overall cohort (n=4,707)


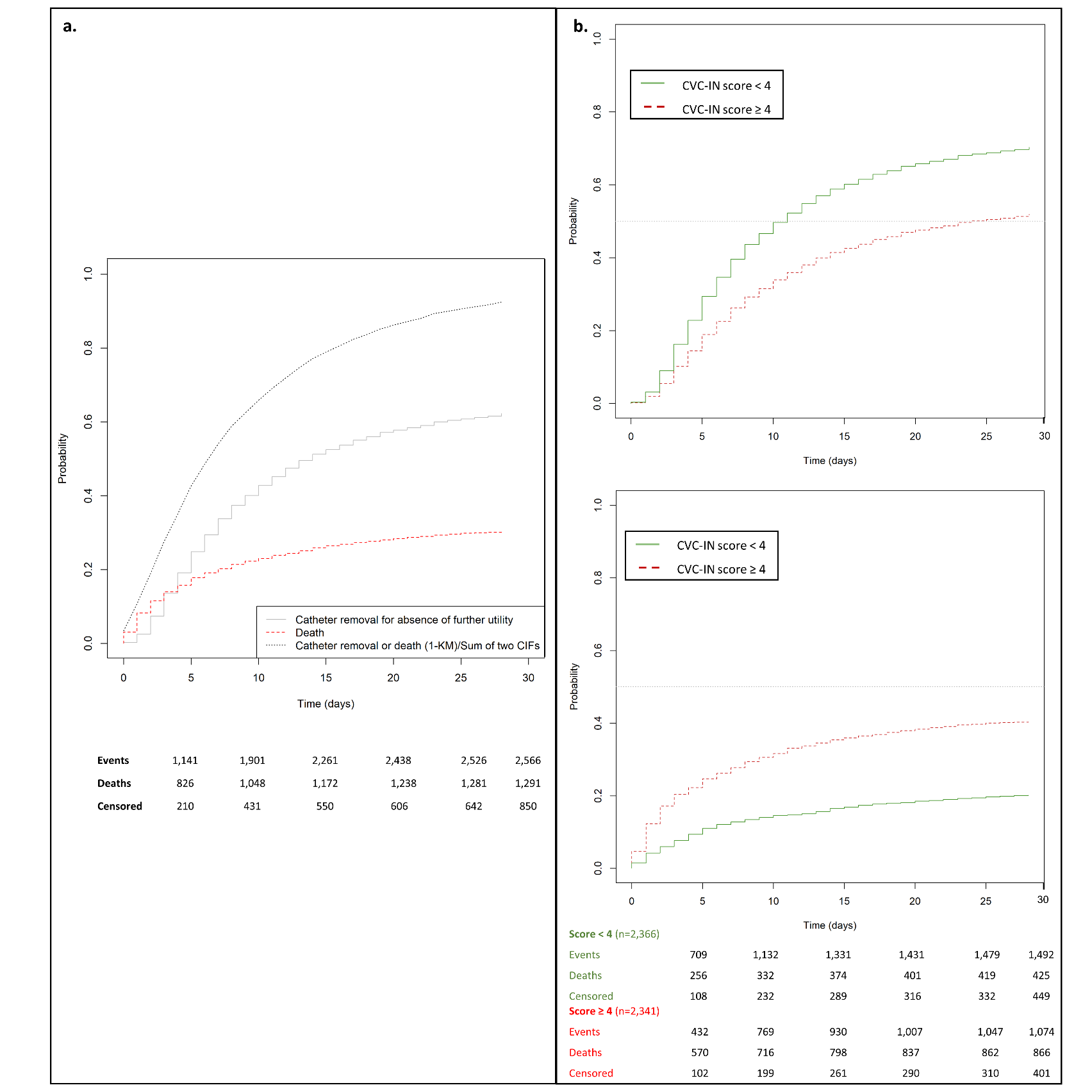


Panel (a.) displays the overall cumulative incidence of catheter removal and death in the overall cohort. Panel (b.) displays the cumulative incidence of catheter removal (top figure) and death (bottom figure) according to the CVC-IN score.

# **Additional file 1: Table S5:** Univariable and multivariable subdistribution hazard models for catheter removal in the training cohort considering only the first catheter in ventilated patients (n=2,336)

|  | **Univariable analysis** | | | **Multivariable analysis** | | |
| --- | --- | --- | --- | --- | --- | --- |
| **Risk factors** | **HR¹** | **95 % CI²** | **p-value** | **Adjusted HR¹** | **95 % CI²** | **p-value** |
| **Male** | 0.90 | [0.81-1.01] | 0.07 | - | - | **-** |
| **Age (years)** |  |  | ***<0.001**** |  |  | ***<0.001**** |
| **< 40** | 1 | - | **-** | 1 | - | **-** |
| **40-59** | 0.65 | [0.54-0.78] | **<0.001** | 0.69 | [0.58-0.84] | **<0.001** |
| **60-69** | 0.54 | [0.44-0.66] | **<0.001** | 0.63 | [0.52-0.77] | **<0.001** |
| **70-74** | 0.50 | [0.39-0.63] | **<0.001** | 0.59 | [0.47-0.75] | **<0.001** |
| **75-80** | 0.43 | [0.34-0.55] | **<0.001** | 0.54 | [0.43-0.68] | **<0.001** |
| **≥ 80** | 0.51 | [0.41-0.64] | **<0.001** | 0.63 | [0.50-0.79] | **<0.001** |
| **Body temperature**  **≥ 39 °C** | 0.95 | [0.77-1.18] | 0.65 |  |  |  |
| **Diabetes** | 0.79 | [0.69-0.91] | **0.002** | - | - | **-** |
| **Obesity** | 0.79 | [0.70-0.91] | **<0.001** | 0.81 | [0.71-0.92] | **0.002** |
| **Vasopressors** | 0.70 | [0.63-0.78] | **<0.001** | 0.79 | [0.71-0.88] | **<0.001** |
| **Immunosuppression** | 0.64 | [0.52-0.78] | **<0.001** | 0.66 | [0.54-0.80] | **<0.001** |
| **Creatinine > 100 µM** | 0.60 | [0.54-0.67] | **<0.001** | 0.67 | [0.59-0.75] | **<0.001** |

*p-value is for type III effect

¹ HR, Hazard-Ratio

² CI, Confidence Interval

# **Additionl file 1: Table S6:** Univariable and multivariable subdistribution hazard models for catheter removal in the training cohort considering the catheters inserted > 72 hours after removal of the previous catheter (n=2,336)

|  | **Univariable analysis** | | | **Multivariable model 1** | | |
| --- | --- | --- | --- | --- | --- | --- |
| **Risk factors** | **HR¹** | **95 % CI²** | **p-value** | **Adjusted HR¹** | **95 % CI** | **p-value** |
| **Male** | 0.89 | [0.80-0.99] | **0.030** | - | - | **-** |
| **Age (years)** |  |  | ***<0.001**** |  |  | ***<0.001**** |
| **< 40** | 1 | - | **-** | 1 | - | **-** |
| **40-59** | 0.65 | [0.54-0.78] | **<0.001** | 0.68 | [0.57-0.82] | **<0.001** |
| **60-69** | 0.53 | [0.44-0.65] | **<0.001** | 0.61 | [0.50-0.74] | **<0.001** |
| **70-74** | 0.48 | [0.38-0.61] | **<0.001** | 0.55 | [0.44-0.70] | **<0.001** |
| **75-80** | 0.44 | [0.35-0.56] | **<0.001** | 0.54 | [0.42-0.68] | **<0.001** |
| **≥ 80** | 0.50 | [0.40-0.62] | **<0.001** | 0.61 | [0.49-0.76] | **<0.001** |
| **Body temperature**  **≥ 39 °C** | 0.95 | [0.77-1.17] | 0.62 |  |  |  |
| **Diabetes** | 0.79 | [0.69-0.91] | **<0.001** | - | - | **-** |
| **Obesity** | 0.87 | [0.77-0.99] | **0.033** | ** | ** | ** |
| **Vasopressors** | 0.69 | [0.62-0.77] | **<0.001** | 0.78 | [0.70-0.87] | **<0.001** |
| **Immunosuppression** | 0.61 | [0.51-0.75] | **<0.001** | 0.65 | [0.53-0.78] | **<0.001** |
| **Creatinine > 100 µM** | 0.59 | [0.53-0.65] | **<0.001** | 0.65 | [0.58-0.73] | **<0.001** |
| **p-value is for type III effect*  ** adjusted hazard-ratio for obesity is 0.89 [0.78-1.01] ; p=0.08  ¹ HR, Hazard-Ratio  ² CI, Confidence Interval | | | | | | |

# **Additoonal file 1: Table S7:** Univariable and multivariable Cox cause-specific models for catheter removal for absence of further utility and death in the training cohort (n=2,336)

|  | **Catheter removal for absence of further utility†** | | | | | |  | **Death‡** | | | | | |
| --- | --- | --- | --- | --- | --- | --- | --- | --- | --- | --- | --- | --- | --- |
|  | **Univariable analysis** | | | **Multivariable analysis** | | |  | **Univariable analysis** | | | **Multivariable analysis** | | |
| **Risk factors** | **CSH¹** | **95 % CI²** | **p-value** | **Adjusted CSH¹** | **95 % CI²** | **p-value** |  | **CSH¹** | **95 % CI²** | **p-value** | **Adjusted CSH¹** | **95 % CI²** | **p-value** |
| **Male** | 0.85 | [0.76-0.95] | **0.004** | 0.89 | [0.79-0.99] | **0.040** |  | 1.04 | [0.87-1.24] | 0.66 |  |  |  |
| **Age (years)** |  |  | ***<0.001**** |  |  | ***<0.001**** |  |  |  | ***<0.001**** |  |  | ***0.002**** |
| **< 40** | 1 | - | **-** | 1 | - | **-** |  | 1 | - | **-** | 1 | - | **-** |
| **40-59** | 0.64 | [0.53-0.77] | **<0.001** | 0.69 | [0.57-0.83] | **<0.001** |  | 1.27 | [0.85-1.89] | 0.26 | 1.11 | [0.74-1.66] | 0.62 |
| **60-69** | 0.53 | [0.43-0.65] | **<0.001** | 0.60 | [0.49-0.73] | **<0.001** |  | 1.47 | [0.98-2.21] | 0.07 | 1.12 | [0.74-1.69] | 0.58 |
| **70-74** | 0.56 | [0.44-0.71] | **<0.001** | 0.62 | [0.49-0.80] | **<0.001** |  | 1.99 | [1.29-3.07] | **0.002** | 1.55 | [1.00-2.40] | **0.049** |
| **75-80** | 0.49 | [0.38-0.62] | **<0.001** | 0.55 | [0.43-0.71] | **<0.001** |  | 1.93 | [1.25-2.98] | **0.003** | 1.30 | [0.84-2.02] | 0.24 |
| **≥ 80** | 0.61 | [0.49-0.76] | **<0.001** | 0.66 | [0.53-0.83] | **<0.001** |  | 2.50 | [1.65-3.77] | **<0.001** | 1.70 | [1.12-2.58] | **0.013** |
| **Temperature ≥ 39 °C** | 0.80 | [0.63-1.01] | 0.07 | - | - | **-** |  | 0.63 | [0.42-0.96] | **0.035** | 0.61 | [0.40-0.93] | **0.022** |
| **Diabetes** | 0.83 | [0.72-0.96] | **0.010** | - | - | **-** |  | 1.21 | [0.99-1.46] | 0.06 | - | - | **-** |
| **Obesity** | 0.75 | [0.65-0.86] | **<0.001** | 0.77 | [0.67-0.88] | **<0.001** |  | 0.82 | [0.67-1.01] | 0.06 | - | - | **-** |
| **Vasopressors** | 0.90 | [0.81-1.01] | 0.07 | - | - | **-** |  | 2.39 | [1.97-2.91] | **<0.001** | 2.04 | [1.67-2.48] | **<0.001** |
| **Immunosuppression** | 0.71 | [0.58-0.87] | **0.001** | 0.72 | [0.58-0.88] | **0.001** |  | 1.75 | [1.41-2.18] | **<0.001** | 1.80 | [1.45-2.24] | **<0.001** |
| **Creatinine > 100 µM** | 0.76 | [0.68-0.85] | **<0.001** | 0.82 | [0.73-0.92] | **<0.001** |  | 2.36 | [1.99-2.81] | **<0.001** | 2.02 | [1.69-2.41] | **<0.001** |

† Hazard-ratios < 1 reflect longer time to catheter removal for absence of further utility. ‡ Hazard-ratios > 1 reflect positive association with mortality.

¹ CSH, cause-specific hazard-ratio ; ² CI, confidence interval
